# Accuracy and reliability of radiological methods for assessing fusion rates in patients undergoing spinal arthrodesis and stabilization: a systematic review of the past 10 years

**DOI:** 10.3389/fsurg.2025.1692887

**Published:** 2025-12-12

**Authors:** Gabriele Bilancia, Deyanira Contartese, Federica Delbello, Giuseppe Tedesco, Francesca Salamanna, Cristiana Griffoni, Alessandro Gasbarrini, Gianluca Giavaresi, Paolo Spinnato

**Affiliations:** 1 Diagnostic and Interventional Radiology, IRCCS Istituto Ortopedico Rizzoli, Bologna, Italy; 2 Surgical Sciences and Technologies, IRCCS Istituto Ortopedico Rizzoli, Bologna, Italy; 3 Department of Rehabilitation Medicine, Spinal Unit, IMFR Gervasutta, Udine, Italy; 4 Department of Spine Surgery, IRCCS Istituto Ortopedico Rizzoli, Bologna, Italy; 5 Department of Biomedical and Neuromotor Science-DIBINEM, University of Bologna, Bologna, Italy

**Keywords:** spinal fusion, arthrodesis, multidetector computed tomography, scoring systems, diagnostic imaging, systematic review

## Abstract

**Background:**

Reliable assessment of spinal fusion remains a significant challenge due to the absence of universally accepted radiological criteria. Despite the widespread use of spinal arthrodesis and stabilization, substantial variability persists in how fusion is defined, assessed, and reported across studies. This systematic review evaluates current radiological methods for assessing spinal fusion outcomes, focusing on their reliability, reproducibility, and clinical applicability, and identifies existing limitations to inform future research and practice.

**Methods:**

A systematic search was conducted in PubMed, Scopus, and Web of Science for studies published between 2014 and 2024. Following PRISMA guidelines, clinical studies reporting explicit radiological criteria for assessing spinal fusion at any vertebral level were included. Extracted data comprised study characteristics, imaging modalities, surgical techniques, fusion definitions, and use of validated scoring systems. Risk of bias was assessed using the ROBINS-I tool.

**Results:**

Of 2,965 articles screened, 557 met the inclusion criteria. Only 36.8% of studies used standardized scoring systems—primarily Bridwell, Brantigan-Steffee-Fraser (BSF), and Lenke classifications. In contrast, 61.2% relied on non-standardized or author-defined criteria, contributing to significant methodological heterogeneity. Computed tomography (CT), alone or combined with conventional radiography (CR), was the predominant imaging method (74.5%), while magnetic resonance imaging (MRI) was used in only 2.0% of studies. Over 200 distinct fusion criteria were identified, underscoring the lack of consensus.

**Conclusions:**

Significant heterogeneity persists in the radiological assessment of spinal fusion, largely due to inconsistent use and interpretation of fusion criteria, even among studies employing established scoring systems. This variability limits comparability across studies and underscores the need for consensus-based, validated guidelines. Future research should prioritize the development and standardization of objective radiological criteria to improve the reliability and clinical applicability of fusion assessment in spinal arthrodesis. Emerging technologies, such as Hounsfield unit–based CT metrics and AI-assisted imaging, appear promising for improving diagnostic accuracy.

**Systematic Review Registration:**

https://www.crd.york.ac.uk/PROSPERO/view/CRD420251111767, PROSPERO CRD420251111767.

## 1 Introduction

Spinal fusion surgery is a widely performed procedure aimed at achieving arthrodesis between two or more vertebrae to restore sagittal balance and spinal stability and alleviating pain associated with various spinal disorders, including degenerative disc disease, deformities, spondylolisthesis, trauma, and tumors (1, 2).

**Figure 1:**
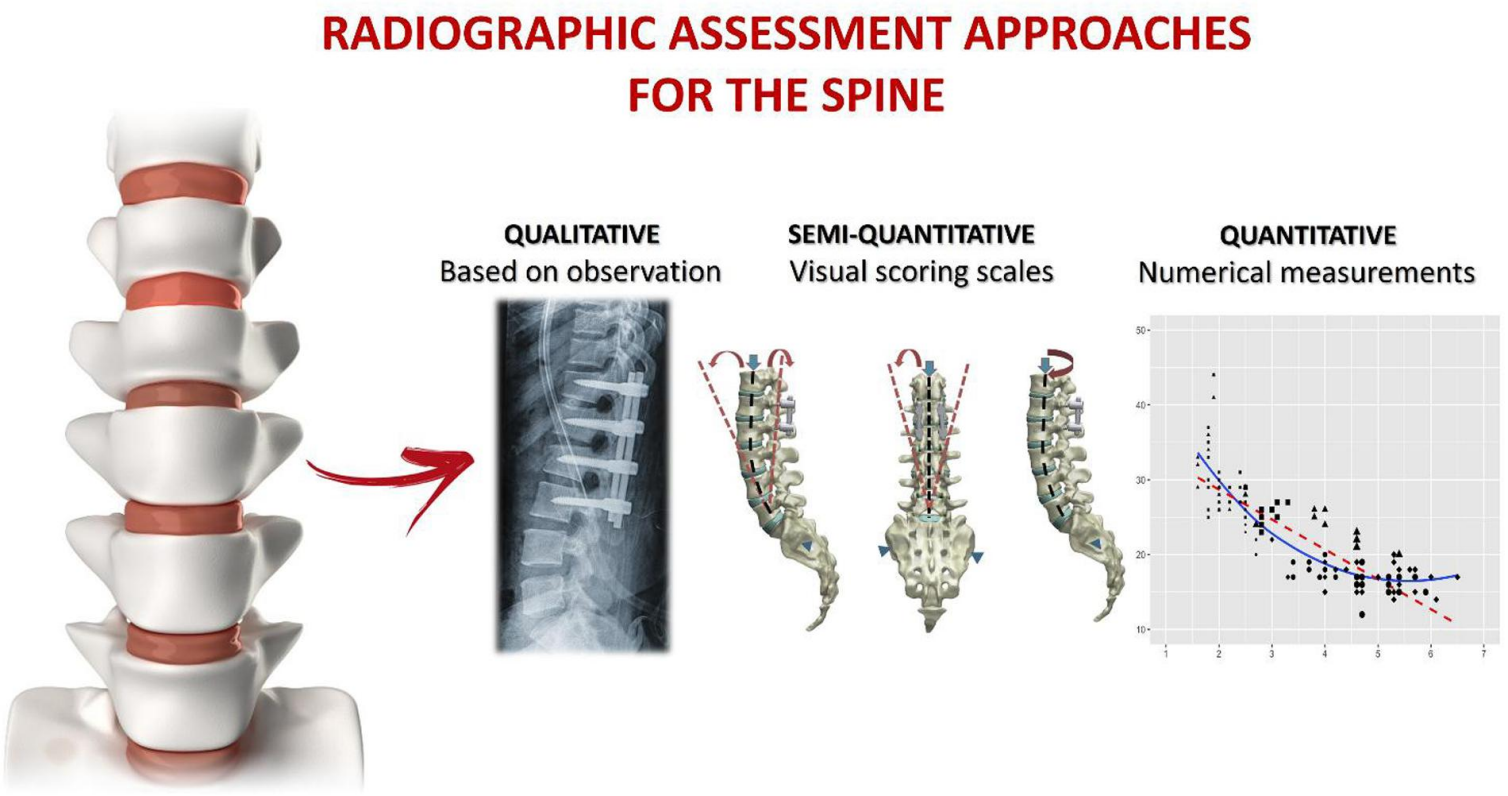
Radiographic approaches for assessing spinal fusion, categorized into three methodological groups. (1) Qualitative methods: assessment based on subjective visual interpretation of imaging findings, including bridging bone, graft incorporation, hardware integrity, or radiolucent lines. (2) Semi-quantitative methods: approaches using structured visual grading systems or dynamic motion criteria, such as flexion–extension angular displacement or validated fusion scoring scales. (3) Quantitative methods: techniques relying on measurable parameters, including densitometric analysis or motion-based thresholds, aimed at providing objective and reproducible evaluation of spinal fusion.

Spinal fusion is performed across all age groups and spinal levels, with the number of procedures increasing annually due to the rising global prevalence of spinal pathologies and aging populations (3). Despite its widespread use and technical evolution, determining the actual success of spinal fusion remains a major clinical challenge in both clinical and research. Postoperative fusion assessment predominantly relies on imaging techniques, yet the choice of diagnostic modality and criteria for defining fusion vary widely across studies and clinical settings (4). Conventional radiography (CR) has traditionally been favoured due to its accessibility and low cost, but computed tomography (CT) is increasingly preferred for its superior ability to visualize bone morphology and the fusion mass (Figure 1). However, fusion rates assessed by CT often diverge significantly from those obtained with CR in the same patient cohorts, complicating the interpretation of surgical outcomes (5). Magnetic resonance imaging (MRI), by contrast, is seldom used because of its limited capacity to accurately depict bone integration.

Beyond imaging modalities, the absence of universally accepted, objective criteria for fusion assessment hinders consistency and comparability of outcomes (6). Several radiological scoring systems, such as the Bridwell grading system, the Brantigan-Steffee-Fraser (BSF) classification, and the Lenke scale, have been proposed to bring structure to fusion evaluation (Figure 1) (7–10). While these frameworks provide a standardized approach, they are constrained by interobserver variability, subjective interpretation, and inconsistent application of grading thresholds (11–13). Moreover, many studies bypass validated scoring systems altogether, relying instead on loosely defined criteria, such as the presence of a continuous fusion mass or the absence of motion on dynamic radiographs, both of which lack reproducibility and uniform clinical interpretation (11). Even with advanced imaging and proposed grading frameworks, methodological heterogeneity remains a key challenge in accurately and consistently assessing spinal fusion.

The lack of consensus on the definition of “successful fusion” has far-reaching implications, directly impacting clinical decisions-making regarding postoperative follow-up, indication for revision surgery, and the evaluation of long-term patient outcomes.

In this context, we conducted a systematic review of the literature published over the past decade to identify existing radiological scoring systems, evaluate the prevalence and consistency of their application, and highlight the need for a standardized and evidence-based framework for spinal fusion assessment. This study aims to synthesize current practices and critically analyze the strengths and limitations of various methodologies, thereby offering valuable insights to guide future research and the development of clinical guidelines in spinal surgery.

## 2 Methods

### 2.1 Eligibility criteria

The PICOS model (Population, Intervention, Comparison, Outcomes, Study design) was used to structure the eligibility criteria for this review: (1) studies involving patients undergoing spine surgery (Population), (2) that included spinal fusion procedures performing arthrodesis and stabilization (Intervention), (3) with or without a comparison group (Comparison), (4) that reported both clinical outcomes of spinal fusion and corresponding radiological findings (Outcomes), (5) and were designed as clinical studies (Study design).

The focused research question was: “*In patients undergoing spinal arthrodesis and stabilization procedures, what qualitative, semi-quantitative, and quantitative methods are used to assess the rate of spinal fusion?*”. Studies published between November 1, 2014, and November 1, 2024, were included if they met the above PICOS criteria. Studies were excluded if they: (1) evaluated surgeries unrelated to the spine, (2) involved patients undergoing spine surgery without spinal fusion, and (3) reported incomplete or missing radiological outcome data that precluded determination of fusion assessment methods. Additionally, the following types of publications were also excluded: reviews, case reports or case series, letters, comments to the Editor, animal studies, *in vitro* studies, pilot studies, meta-analyses, editorials, protocols and recommendations, guidelines, and articles not written in English.

### 2.2 Search strategies

Current literature review involved a systematic search conducted in November 2024 and was performed in accordance with the Preferred Reporting Items for Systematic Reviews and Meta-Analyses (PRISMA) statement (14). This review was registered at PROSPERO (ID: CRD420251111767). The search was conducted across three major databases: PubMed, Scopus, and Web of Science. The following combination of terms was used: (spine disease OR spine surgery) AND (spinal fusion OR arthrodesis OR fusion assessment OR radiological evaluation OR fusion grading). For each of these concepts, both free-text terms and controlled vocabulary specific to each bibliographic database (e.g., MeSH terms for PubMed) were used and combined using the operator “OR”. The terms themselves were then combined using “AND”. The complete search strategies, including the combinations of free-text and controlled vocabulary terms used in PubMed, Scopus, and Web of Science, are detailed in Supplementary Table S1 (Supplementary Materials, Section A1).

### 2.3 Selection process

The data selection and management process for this systematic review was conducted using the Rayyan platform, a specialized tool designed to streamline the organization and screening of citations retrieved from multiple scientific search engines. Rayyan was employed to facilitate the initial screening based on titles and abstracts, followed by full-text analysis of potentially relevant articles. The platform also enabled the identification and removal of duplicate records, ensuring a clean and accurate set of publications for final inclusion in the review.

After importing the retrieved articles into the systematic review software Rayyan to remove duplicates, potentially relevant studies were screened by three independent reviewers (GB, DC, and FD) based on titles and abstracts. Studies that did not meet the inclusion criteria were excluded. Any disagreements during the screening process were resolved through discussion to reach a consensus. If consensus could not be achieved, a fourth reviewer (PS) was consulted to make the final decision. The remaining studies that passed the screening phase were included in the final stage of data extraction.

### 2.4 Data extraction and synthesis

The data extraction and synthesis process began with cataloguing the study details. To increase validity and avoid omitting potentially relevant findings for the synthesis, three authors (GB, DC, and FD) independently extracted data and compiled it into a Database (Supplementary Materials, Excel file), considering the following aspects: study type, number of patients, age and gender, medical condition, comorbidities, type of surgery, presence of posterior fixation, implant type (cage, bone graft, bone substitute) and design (radiopaque, radiolucent), imaging methods (CR, CT, MRI), spinal fusion region (cervical, thoracic, lumbar, sacral), number of fused levels (single or multiple), fusion score, fusion criteria, fusion values, sign of nonunion, radiographically extracted parameters pertaining to fusion evaluation, fusion outcomes, follow up duration, presence of comparison groups, and study results.

### 2.5 Assessment of methodological quality

Three reviewers (GB, DC, and FD) independently analyzed the methodological quality of the included studies. In case of disagreement, they attempted to reach a consensus; if consensus was not achieved, a fourth reviewer (PS) made the final decision. The methodological quality of the included clinical studies was assessed using the Cochrane Risk of Bias in Non-randomized Studies of Interventions (ROBINS-I) tool (15). This tool for nonrandomized trials included seven domains that assess possible sources of bias: bias due to confounding, bias in the selection of participants into the study, bias in the classification of interventions, bias due to deviations from intended interventions, bias due to missing data, bias in the measurement of outcomes, and bias in the selection of the reported result. Each domain was assigned one of three levels: low risk of bias, moderate risk of bias, or high risk of bias, until an overall risk of bias judgment was reached.

### 2.6 Data analysis

Once the eligible studies were selected, all relevant data were systematically extracted and compiled into a dedicated Microsoft Excel database, structured to support consistent and comprehensive data analysis. This database included key variables, methodological characteristics, and outcome measures across the reviewed literature. Furthermore, all figures and graphical representations used in the study were created directly within Excel, allowing for clear visualization of trends, distributions, and comparative metrics across the included articles.

## 3 Results

### 3.1 Study selection and characteristics

**Figure 2:**
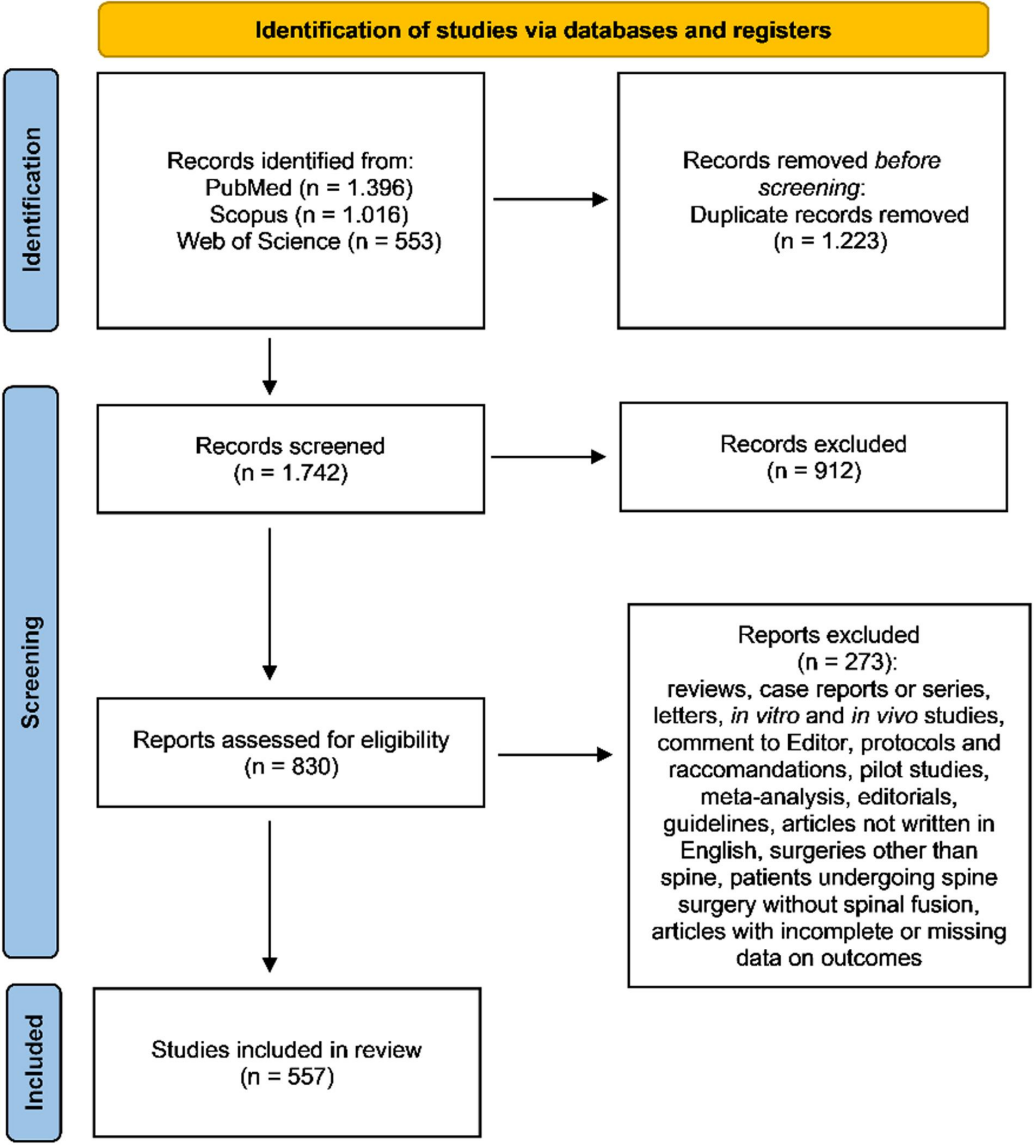
PRISMA (Preferred Reporting Items for Systematic Reviews and Meta-Analyses) flow diagram illustrates the systematic review process, including details of database searches, number of abstracts screened, full-text articles assessed for eligibility, and final studies included.

The initial literature search retrieved 2,965 studies. Of those, 1,396 were identified through PubMed, 1,016 through Scopus, and 553 through Web of Science. The articles were then uploaded to a public reference manager to remove duplicates. After duplicate removal, 1,742 articles remained and were screened by title and abstract, resulting in 830 articles selected for full-text review to determine eligibility. Ultimately, 557 articles met the inclusion criteria and were included in this review. Among these, 377 were retrospective cohort studies, and 180 were prospective cohort studies. The search strategy, as well as the study inclusion and exclusion criteria, are detailed in Figure 2.

### 3.2 Risk of bias assessment

**Figure 3:**
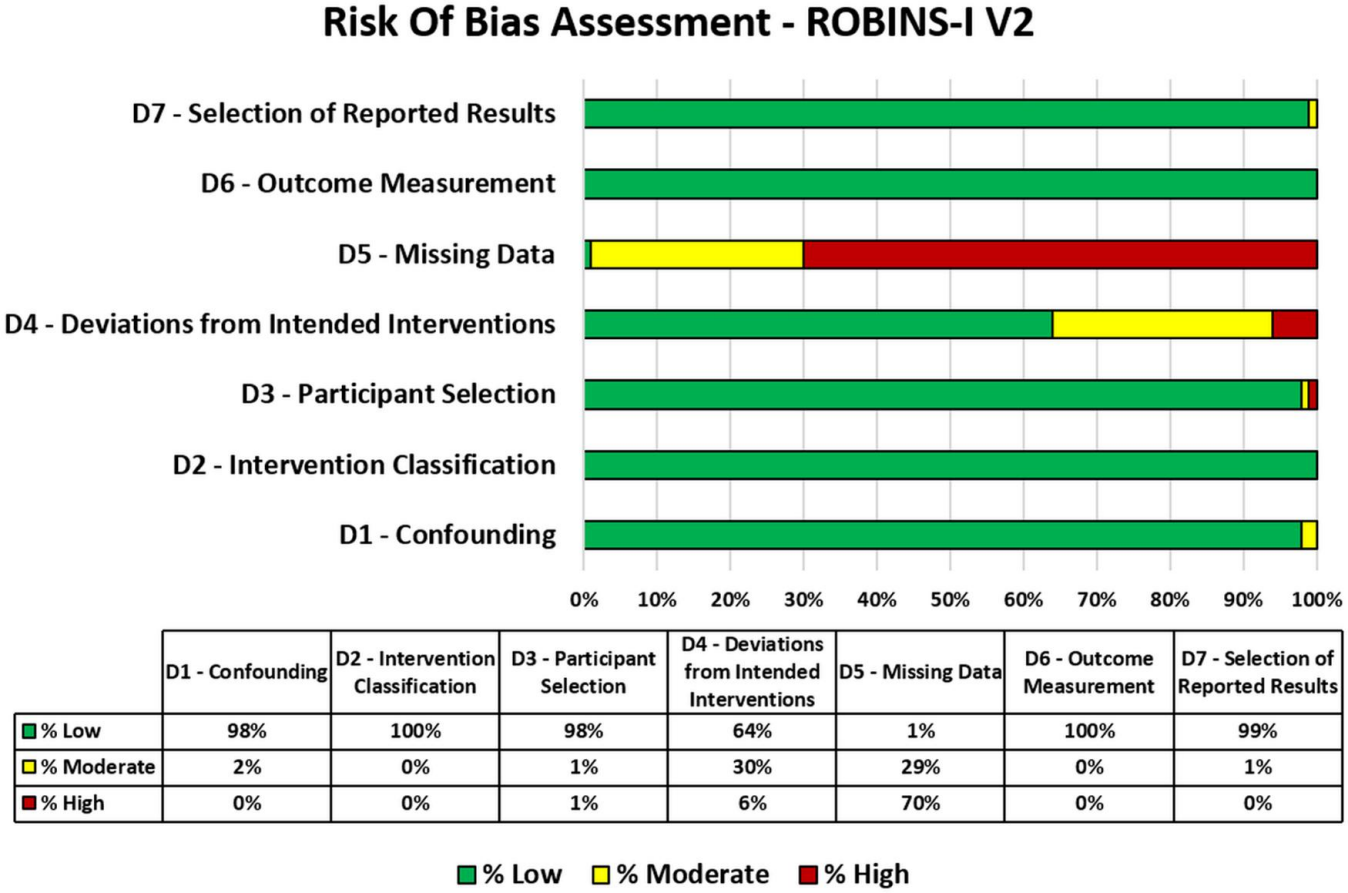
Risk of bias assessment of included studies using the ROBINS-I (Risk of Bias in Non-randomized Studies of Interventions, version 2) tool. The figure illustrates the proportion of studies rated as low, moderate, serious, or critical risk of bias across key methodological domains.

The assessment of risk of bias for the clinical trials included in this review is presented in Figure 3. The evaluation across the seven ROBINS-I domains revealed considerable variation in methodological quality among the included studies. Most domains—such as confounding, intervention classification, participant selection, outcome measurement, and selection of reported results—consistently showed a low risk of bias, indicating rigorous control in these areas. However, notable weaknesses were identified in the domains of missing data and deviations from intended interventions. Specifically, missing data was the most problematic domain, with most studies (*n* = 390) exhibiting a high risk of bias, highlighting potential threats to internal validity. Deviations from intended interventions were associated with moderate to high levels of bias, often reflecting variability in how interventions were implemented and monitored across studies.

### 3.3 Studies results

#### 3.3.1 General information's

**Figure 4:**
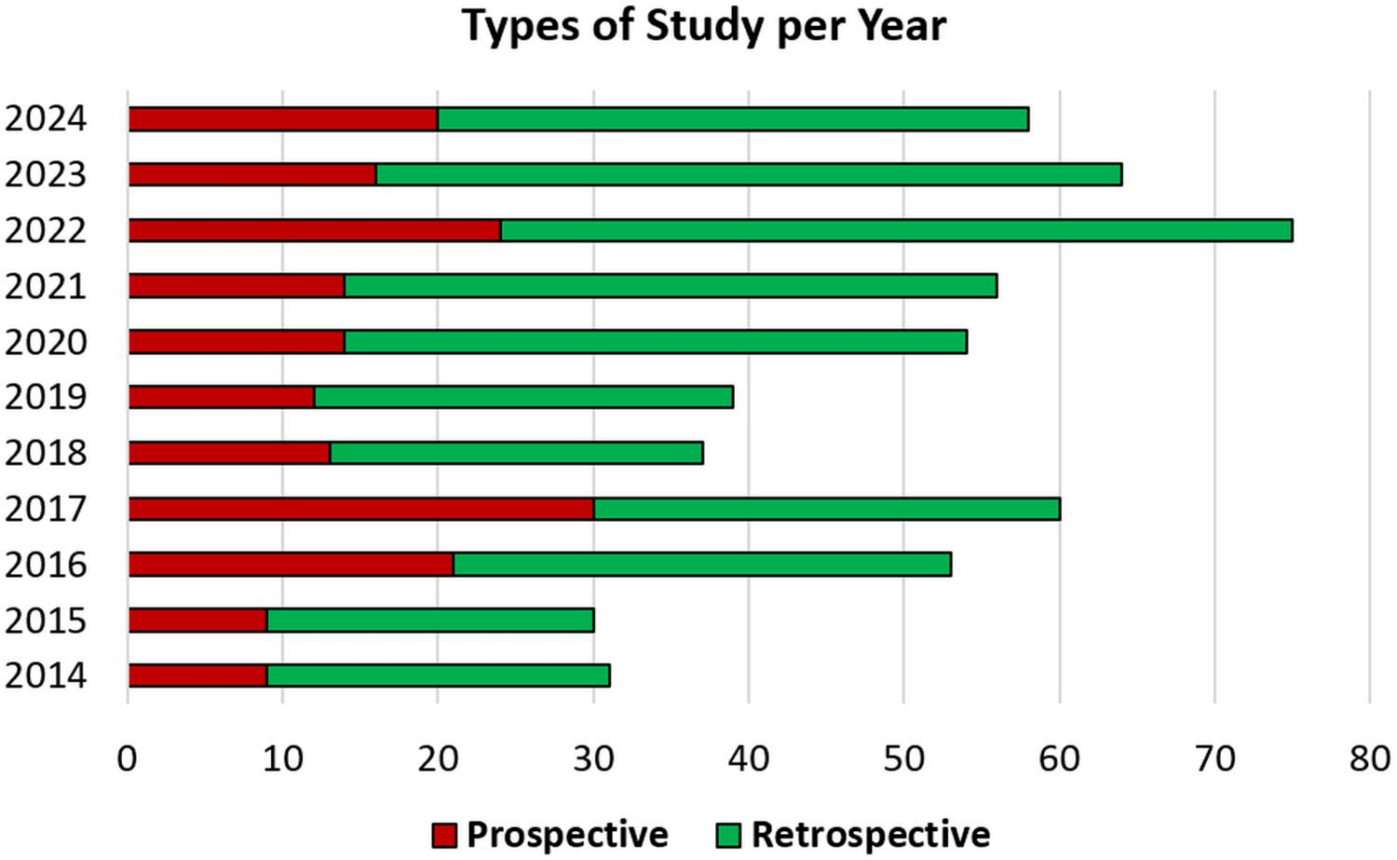
Annual distribution of prospective and retrospective studies published from 2014 to 2024, demonstrating a progressive increase in research output and underscoring the growing interest in spinal fusion evaluation.

The annual distribution of included studies published between 2014 and 2024 is illustrated in Figure 4. The data show a consistent increase in the number of studies over time, reflecting a growing research interest in spinal fusion and its evaluation.

A total of 49,694 patients undergoing spinal fusion procedures were analyzed across the included studies. The mean patient age was 57.5 years, ranging from 25 to 93 years. The median follow-up duration was 22.5 months (range: 6–168 months), with a consistently high fusion rate reported in all studies. Several comorbidities were commonly reported, including heart failure, hypertension, alcoholism, osteoporosis, diabetes, obesity, and mental illness.

#### 3.3.2 Surgical procedures and clinical indications

Spinal procedures included surgeries of varying complexity, categorized as minor, major, and complex. These comprised arthrodesis, corpectomy, microdiscectomy, decompression, laminectomy, laminoplasty, open and minimally invasive interbody fusion techniques, including posterior lumbar interbody fusion (PLIF), transforaminal lumbar interbody fusion (TLIF), oblique lumbar interbody fusion (OLIF), anterior lumbar interbody fusion (ALIF), lateral lumbar interbody fusion (LLIF), anterior cervical discectomy and fusion (ACDF), and anterior cervical corpectomy and fusion (ACCF). Spinal fusion was applied to a wide range of spinal diseases, primarily degenerative conditions such as disc herniation, stenosis, spondylolysis, radiculopathy, spondylolisthesis, and adult spinal deformity.

To improve clarity and usability of the collected data, the clinical conditions leading to spinal arthrodesis were grouped into seven main categories based on the spinal region involved. An additional subcategory was created for the cervical spine to specifically address myelopathies and related conditions, highlighting the distinct clinical relevance of this group. The main categories were degenerative diseases, herniated disc and recurrence, spinal stenosis, spondylolisthesis, spondylolysis and instability, spinal deformities, other specific or rare diseases, and unclear diseases (used when the underlying pathology was not explicitly stated). Surgical approaches were similarly categorized into macro-categories according to spinal region (cervical, thoracic, and lumbar) allowing a structured comparison and systematic description of treatment trends across different anatomical sites. None of the included studies focused exclusively on the sacral region.

A detailed overview of regional trends in surgical practice, including the anatomical distribution of spinal fusion procedures and associated clinical indications, is provided in the Supplementary Materials (Section A2).

#### 3.3.3 Instrumentation and materials

**Figure 5:**
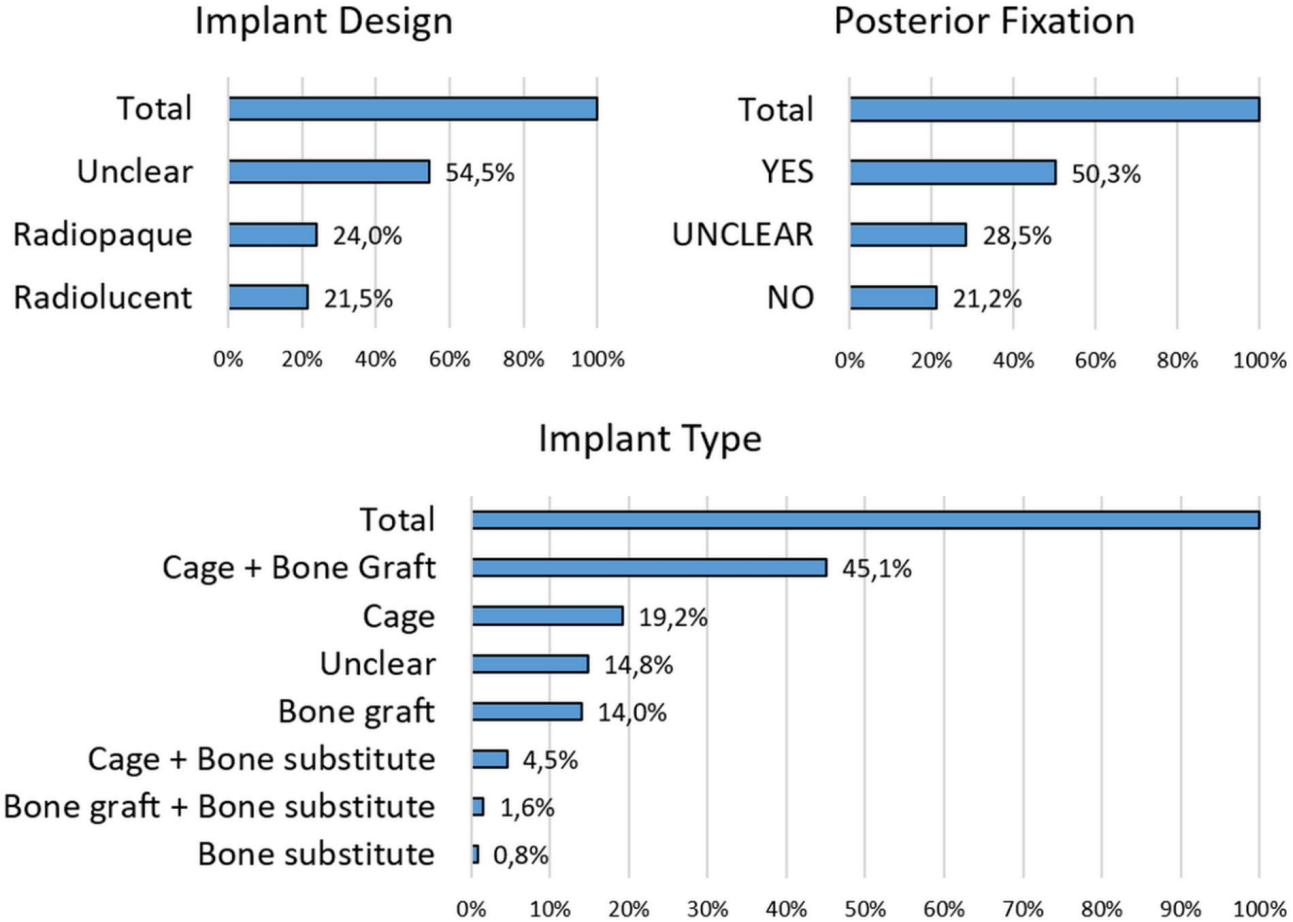
Distribution of implant characteristics used in spinal fusion procedures across the included studies. The total number of cases was normalized to 100%.

The analysis provided detailed information on the surgical approaches reported in the included studies. A primary focus was on the use of posterior fixation with screws and rods, categorized as “Posterior Fixation: YES, NO, or UNCLEAR”, as part of the spinal arthrodesis procedure. As illustrated in Figure 5, the total number of cases was normalized to 100%. Among these, 50.3% involved posterior fixation (YES), 21.2% did not (NO), and in 28.5% of cases, the fixation status was not specified (UNCLEAR). These findings indicate that posterior fixation was reported in most of cases, while a substantial portion of studies did not clearly document fixation status. Materials used to achieve fusion were documented and classified into the following categories: cage, bone graft, cage + bone graft, bone substitute, cage + bone substitute, bone graft + bone substitute, and unclear. Additionally, studies were assessed based on whether the interbody cages used were radiolucent or radiopaque. The corresponding data are presented in the charts below (Figure 5). The analysis reveals a clear trend toward the use of radiopaque materials (24.0% of studies), particularly when combined with interbody cages and bone grafts (45.1%). This combination was the most frequently reported in the included studies.

#### 3.3.4 Imaging techniques used in spinal fusion assessment

**Figure 6:**
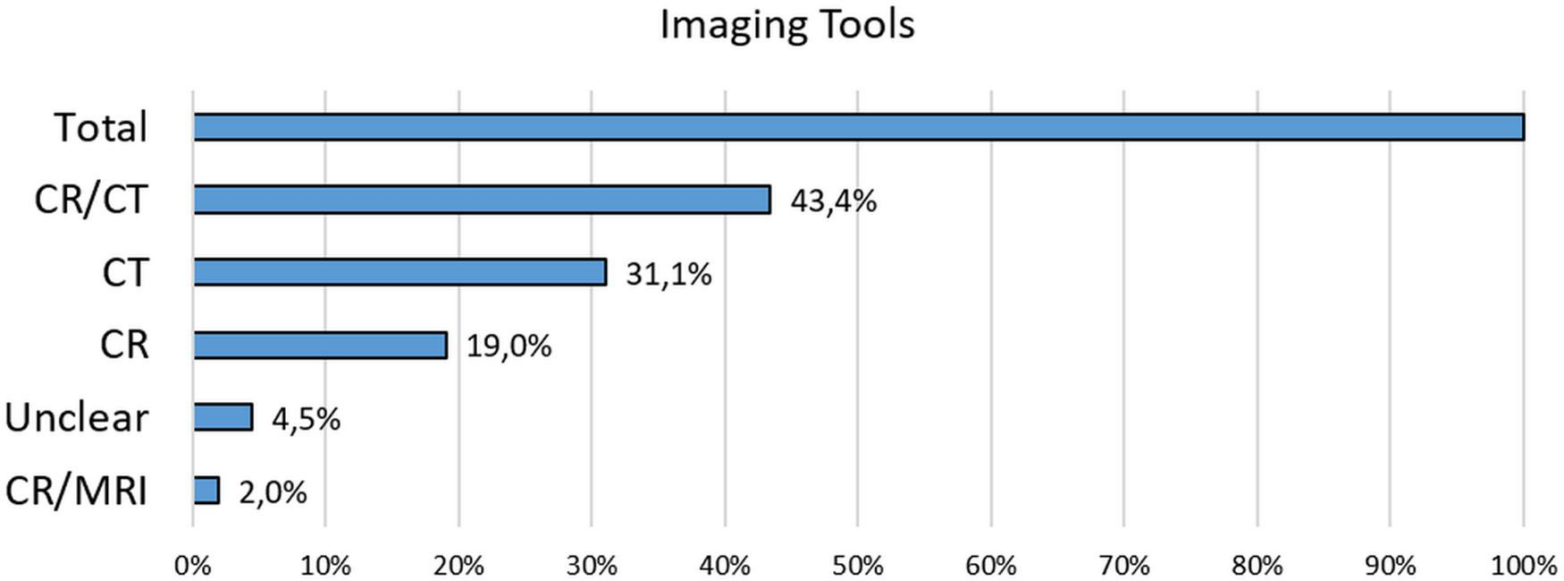
Distribution of imaging modalities used to assess spinal fusion. The total number of cases was normalized to 100%. CR: Conventional radiography; CT: computed tomography; MRI: Magnetic resonance imaging.

The data presented in the accompanying chart (Figure 6) clearly indicates that the most employed imaging modality for assessing spinal fusion is the combined use of CR and CT scans (CR/CT). This is followed by CT alone and CR alone, reinforcing the role of CT as the primary tool for postoperative monitoring of spinal fusion, due to its superior ability to visualize bone structures and detect fusion-related changes.

MRI alone was used in only 2.0% of studies, reflecting its limited utility for assessing bone fusion, likely due to lower spatial resolution and reduced sensitivity to bone density. The “Unclear” category includes studies in which the imaging modality used to assess fusion was not clearly reported.

#### 3.3.5 Fusion scoring systems

The analysis of the included studies showed that only 36.8% used a widely recognized scoring system to evaluate spinal fusion. In contrast, 61.4% did not employ any standardized criteria, indicating considerable variability in fusion assessment methods. The “Unclear” category (2.0%) includes studies in which the method of fusion assessment could not be determined from the available information.

**Figure 7:**
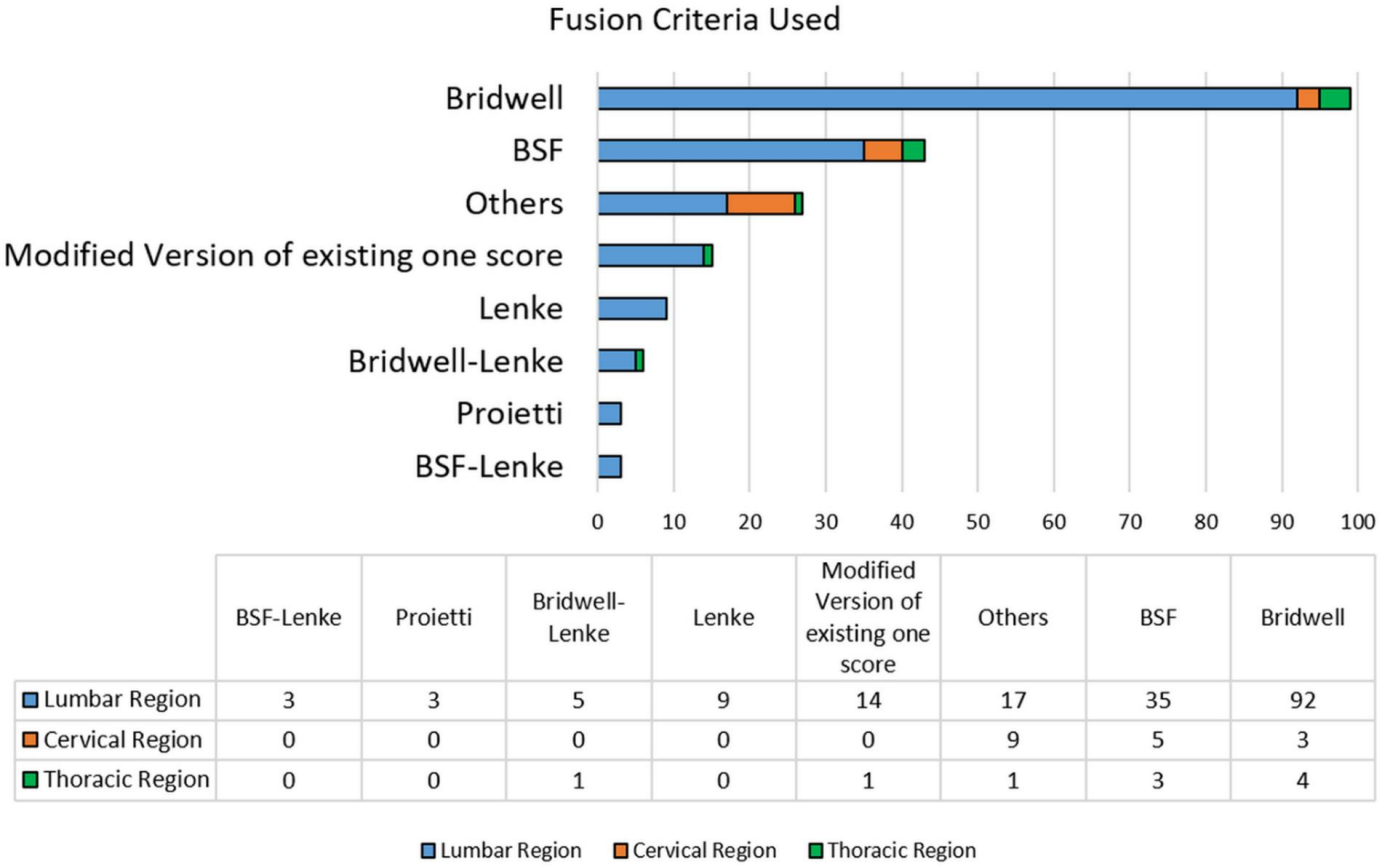
Detailed breakdown of the fusion scoring systems utilized in the reviewed studies. The *X*-axis and table values indicate the number of studies reporting each scoring system. BSF: Brantigan-Steffee-Fraser.

Among the standardized systems, the Bridwell score was the most frequently applied, followed by the BSF score. Other systems, including Lenke, Bridwell-Lenke, and BSF-Lenke, were used less often (Figure 7).

The “Others” category encompasses scoring methods reported in the literature but cited in only a single study, suggesting limited adoption, lack of standardization, and insufficient validation. A notable proportion of studies used modified versions of established scoring systems, often without providing methodological justifications (Figure 7), which may affect reproducibility and comparability with studies using original versions. In some cases, multiple scoring systems were applied within the same study. While this may reflect an attempt to enhance assessment accuracy, it also introduces concerns regarding methodological consistency and can hinder data interpretation.

#### 3.3.6 Alternative definitions of fusion in the absence of standardized scoring systems

**Figure 8:**
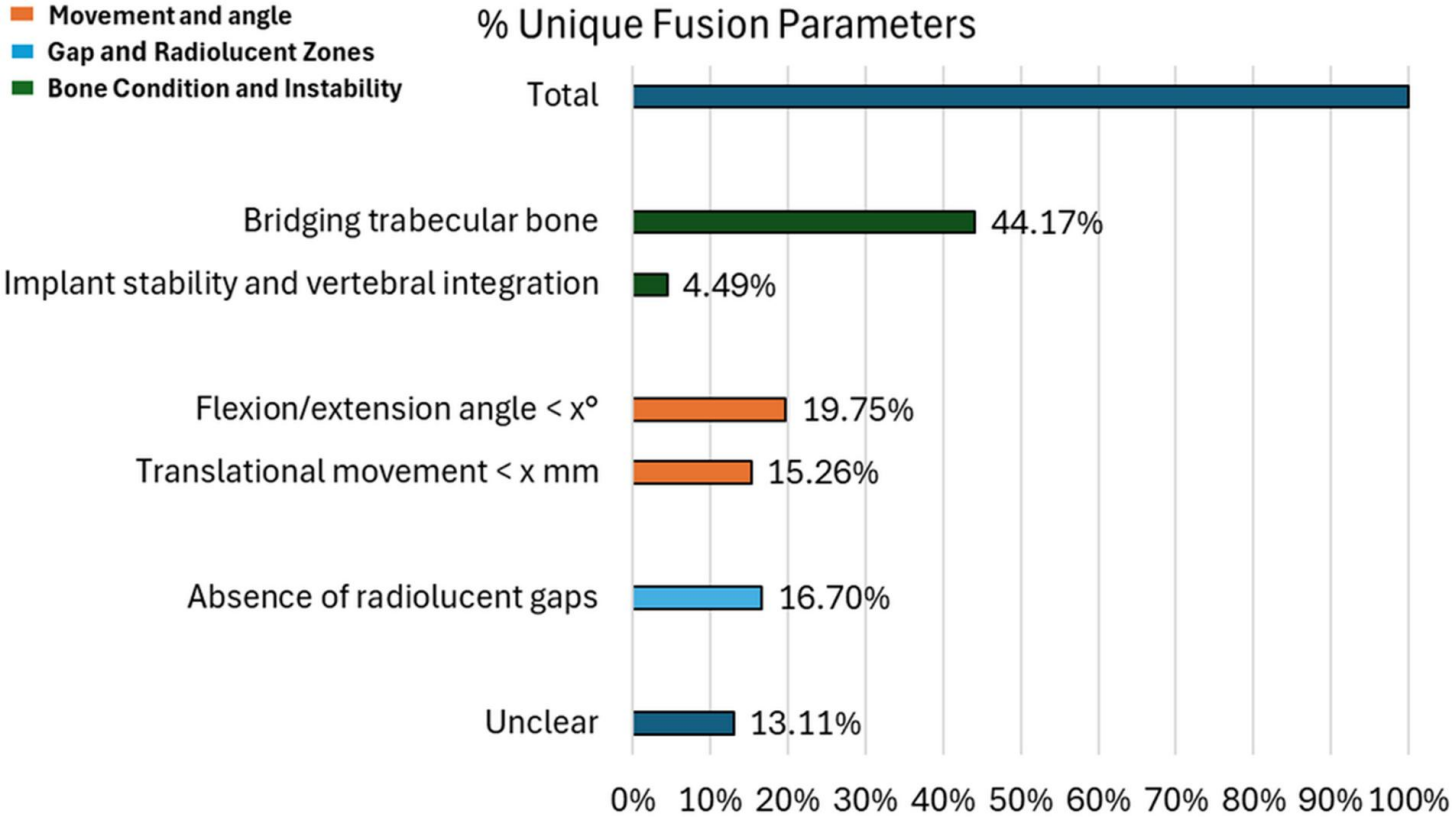
Percentage distribution of distinct evaluation parameters used to define spinal fusion across the included studies. The total number of cases was normalized to 100%. Thresholds indicated as “x°” for flexion/extension angle and “x mm” for translational movement refer to generic cut-off values founded in the analyzed articles.

Among studies that did not employ a specific scoring system for spinal fusion, a total of 205 distinct definitions of fusion were identified. Although many of these definitions shared common variables, they varied significantly in the threshold values used to define successful fusion (Figure 8). The primary criteria observed included: 1) Movement and Angle—assessing intersegmental motion and postoperative angular variation; 2) Gap and Radiolucent Zones—evaluating the presence of intervertebral gaps and radiolucent areas, which may indicate incomplete fusion; 3) Bone Condition and Instability—examining the quality of the fusion mass and the mechanical stability of the operated segment. This wide variability in threshold values across studies underscores a pronounced heterogeneity in fusion assessment methodologies, which may compromise comparability and consistency of outcomes across the literature. Among the identified indicators, the most frequently reported was the presence of continuous trabecular bone bridging, highlighting its perceived importance as a hallmark of successful fusion.

Beyond the primary criteria outlined above, several additional findings emerged. First, implant stability and vertebral integration were rarely used as criteria for movement and angulation, suggesting that mechanical factors are generally considered less important than bone-related markers. Second, some definitions included the absence of radiolucent gaps, highlighting an emphasis on cortical continuity. Third, translational motion and flexion/extension angle were evaluated in a minority of definitions, indicating that while residual mobility is acknowledged, it is not widely regarded as a primary determinant of fusion. Finally, a substantial proportion of definitions remained unclear, illustrating a persistent lack of clarity and standardization in the reporting of fusion criteria (Figure 8).

Among all evaluated variables, the greatest heterogeneity was observed in the threshold values applied to translational motion (e.g., <x mm) and flexion/extension angle (e.g., <x°). As detailed in Table 1, 114 articles reported thresholds for flexion/extension angles. The most used value was 5° (cited in 35 articles), followed by 2° (27 articles), 3° (19 articles), 4° (15 articles), and 10° (1 article). In 17 studies, the threshold angle was not specified. For translational movement, 82 articles provided thresholds, with 3 mm being the most frequently cited value (33 articles), followed by 1 mm and 2 mm (18 articles each), and 4 mm (1 article). Thresholds were unspecified in 12 articles. However, in many cases, these thresholds lacked a methodological justification, rendering their clinical application vulnerable to subjective interpretation. This lack of standardization introduces a potential source of bias in both the evaluation and reporting of spinal fusion outcomes.

**Table 1:** Reported threshold values for flexion/extension angles and translational movement used to assess spinal fusion across included studies. Thresholds indicated as “x°” for flexion/extension angle and “x mm” for translational movement refer to generic cut-off values founded in the analyzed articles.

Flexion/extension angle <x°	% of articles	Translational movement <x mm	% of articles
2°	4.8 (*n* = 27)	1	3.2 (*n* = 18)
3°	3.4 (*n* = 19)	2	3.2 (*n* = 18)
4°	2.7 (*n* = 15)	3	5.9 (*n* = 33)
5°	6.3 (*n* = 35)	4	0.2 (*n* = 1)
10°	0.2 (*n* = 1)	Not Specified	2.2 (*n* = 12)
Not specified	3.1 (*n* = 17)	—	—
**Total**	**20.5 (*n* = 114)**	**Total**	**14.7 (*n* = 82)**

The “Total” rows indicate the cumulative proportion and number of articles reporting thresholds for flexion/extension angles (20.5%, *n* = 114) and translational movement (14.7%, *n* = 82).

#### 3.3.7 Use of fusion rate as a comparative outcome measure

**Figure 9:**
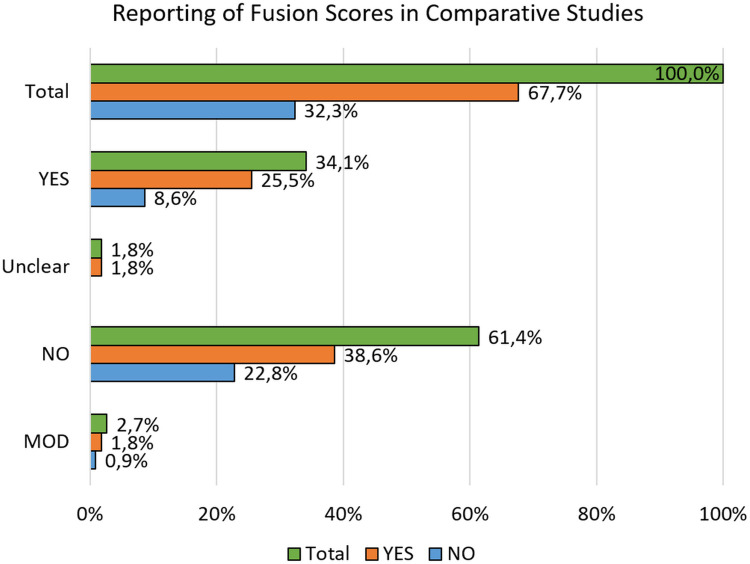
Reporting of fusion scores in comparative studies. MOD: modified version of the original scoring system.

To gain insight into methodological consistency, an assessment was conducted to determine whether the included studies employed formal scoring systems for spinal fusion evaluation. Studies were grouped based on whether they used a validated scoring system (“YES”), lacked any defined scoring method (“NO”), employed a modified version of an existing score (“MOD”), or had an unspecified methodology (“Unclear”). These categories were then assessed to see if fusion rate was used to compare surgical techniques or biomaterials. Among the 557 studies analyzed, 191 studies clearly reported a specific fusion scoring system, and of these, 142 incorporated fusion rates as a comparative outcome (Figure 9). In contrast, 342 studies did not utilize any scoring tool: notably, 215 of these still used fusion rates as a basis for methodological comparison. A smaller group of 15 studies employed a modified scoring system, with 10 of these using fusion rate as a discriminant. Finally, 10 studies provided unclear scoring criteria, yet all of them applied fusion rate in their comparative analyses.

These findings indicate that a substantial number of studies rely on fusion rate as a comparative outcome despite not applying any standardized or validated criteria to define or assess fusion (Figure 9). This highlights a key methodological inconsistency, as the interpretation of fusion rate may vary depending on subjective judgment or undefined parameters.

Even among studies that report using formal scoring systems, variation persists in the selected criteria, scoring scales, and modifications applied. This reflects a broader and persistent issue in spinal fusion research: the lack of a universally accepted definition or measurement standard for spinal fusion. This absence undermines the comparability of findings across studies and call into question the clinical validity of fusion rate as a standalone indicator of treatment success.

## 4 Discussion

Spinal fusion remains a foundational procedure in the treatment of degenerative spine disease, with its efficacy reliant on reliable assessment methods to confirm arthrodesis. Yet, this review highlights a methodological fragmentation and lack of consensus in how fusion is defined and evaluated. Across the 557 studies included, more than 200 different criteria were identified, reflecting substantial heterogeneity in evaluation approaches. Widely used diagnostic parameters, such as intersegmental angular motion thresholds, cortical continuity, and radiolucent gap identification, are applied inconsistently across studies, often without evidence-based justification (4, 11, 16). Such variability undermines the reproducibility and clinical applicability of published data (11).

CT is the most frequently employed modality due to its high spatial resolution and capacity to visualize fine osseous structures (4, 5, 11). In prior studies, CT was used in 62%–75% of spinal fusion assessments, whereas plain radiography accounted for 40%–60% (4, 5). Despite this widespread use, most studies rely on qualitative or semi-quantitative scoring, contributing to interobserver variability. Fine-cut reconstructions enhance imaging accuracy (5), yet many studies rely on subjective interpretation, contributing to interobserver variability and diagnostic ambiguity. MRI, with its limitations in bone imaging, is rarely utilized (4). Moreover, a substantial proportion of studies lack a clear description of imaging protocols (11), pointing broader concerns regarding methodological transparency.

Comparisons with previous reviews reveal both consistencies and discrepancies. Lehr et al. identified a similar lack of standardized imaging criteria, noting that only 30% of studies used quantitative thresholds, consistent with our findings (11). In contrast, Yu et al. reported slightly higher adoption of semi-quantitative scoring (45%), reflecting recent trends toward more structured evaluations (13). These differences likely stem from variations in sample sizes, vertebral levels studied, and types of implants used.

The absence of a unified, standardized scoring system for assessing spinal fusion represents a central challenge—and the primary focus of this analysis. This lack of methodological consensus, compounded by the limited correlation with clinical outcomes such as disability indices and health-related quality of life metrics (17–19), continues to hinder the development of robust, evidence-based guidelines. Although professional associations have introduced surgical protocol frameworks (1, 2, 6), a radiologically validated and universally accepted definition of fusion remains elusive (4).

In this context, densitometric quantification via Hounsfield Units (HU) derived from CT imaging emerges as a promising methodological alternative. HU analysis offers an objective and reproducible, measure of bone mineralization and has demonstrated encouraging correlation with fusion grading scales and clinical outcomes (10). The application of defined HU thresholds allows for consistent evaluation across patient cohorts, operative techniques, and biomaterials (20, 21). However, our review did not include direct head-to-head comparisons of HU-based vs. conventional qualitative methods; therefore, claims of superiority remain speculative. Variability in fusion assessment may be partially explained by differences in regions of interest (ROI) selection, imaging parameters, and postoperative timing. Moreover, target segmentation of ROI, combined with semi-automatic processing tools, enhances both efficiency and cross-study comparability, key steps toward broader clinical integration (4). The increasing use of radiolucent implants, such as those made from CFR-PEEK, further supports HU-based assessments by improving image clarity and minimizing metallic artifact interference—an essential consideration in quantitative fusion analysis (22, 23). Moreover, advances in biomaterials, including structural allografts and engineered interbody implants, further underscore the need for precise and reproducible monitoring of fusion status (7, 8, 24, 25). Metallic cages, in contrast, can obscure bone detail and potentially introducing bias. The increasing heterogeneity of implant types across studies introduces additional sources of methodological bias, reinforcing the critical need for standardized, objective criteria in both clinical and research settings (11).

Looking forward, the integration of HU metrics into artificial intelligence (AI) models represents a promising avenue for overcoming current limitations. AI applications in spine imaging have demonstrated potential for automated grading, outcome prediction, and decision support (26, 27). When trained on annotated HU datasets, deep learning algorithms could redefine fusion diagnostics by reducing subjectivity and enabling a measurable, reproducible approach to postoperative assessment. However, these approaches remain investigational and require validation across diverse patient cohorts.

Nonetheless, several limitations of the current evidence base must be acknowledged. Many included studies suffer from small sample sizes, lack multicentre validation, and offer limited evaluation of interobserver agreement (4, 11, 13). These constraints restrict generalizability and underscore the need for large-scale trials adhering to rigorous methodological frameworks such as PRISMA and ROBINS-I (14, 15). The risk of bias assessment revealed generally strong methodological quality across several domains, including confounding, intervention classification, and outcome measurement. However, high risk of bias due to missing data was a major concern, affecting most studies and threatening internal validity. Additionally, deviations from intended interventions introduced moderate to high bias, reflecting inconsistencies in protocol adherence. These findings highlight the need for improved data management and intervention standardization in future research. An important aspect highlighted by this review is the need to understand the mechanisms underlying the substantial variability observed across studies in the radiological assessment of spinal fusion. Several interacting factors contribute to this heterogeneity. First, biomechanical differences among spinal levels, patient-specific anatomical variations, and distinct biological healing capacities affect the progression and radiographic appearance of fusion. These biological variables can lead to inconsistencies in traditional morphological markers of arthrodesis, such as cortical continuity, trabecular bridging, or reduction in intersegmental motion. Second, technical factors—including variability in CT slice thickness, reconstruction algorithms, x-ray magnification, patient positioning, and implant materials—directly influence the visibility and interpretation of fusion-related features. For example, metal-induced artifacts may obscure early bone formation, whereas the increasing use of radiolucent implants can enhance the apparent fusion mass, potentially inflating diagnostic confidence. Finally, heterogeneous timing of postoperative imaging further complicates interpretation: early imaging may capture transient inflammatory or remodelling phases, whereas delayed imaging may fail to distinguish between solid fusion and stable pseudoarthrosis. These biological, technical, and temporal factors collectively contribute to the inconsistent reporting of fusion rates in the literature and underscore the need for harmonized imaging protocols and objective, quantitative criteria.

In summary, this review confirms the wide heterogeneity in spinal fusion assessment and highlights the urgent need for objective, reproducible, and standardized evaluation methods. By situating these findings within the existing literature and considering underlying methodological factors, we provide a more comprehensive understanding of current limitations and future directions in spinal fusion research.

## 5 Conclusion

Spinal fusion assessment remains highly variable, with over 200 different criteria identified across the literature, limiting reproducibility and comparability. Despite widespread use of radiography and CT, current evaluations often rely on qualitative or inconsistently applied scoring systems. This review highlights the lack of standardized, objective criteria for fusion assessment and emphasizes the need for future studies to develop validated, evidence-based guidelines. Standardizing fusion assessment would improve comparability across studies and support more consistent clinical decision-making.

## Data Availability

The original contributions presented in the study are included in the article/Supplementary Material, further inquiries can be directed to the corresponding author.
