# Supplementary material for: Accuracy and reliability of radiological methods for assessing fusion rates in patients undergoing spinal arthrodesis and stabilization: a systematic review of the past 10 years
Source: Front Surg. 2025 Dec 12;12:1692887. doi: 10.3389/fsurg.2025.1692887 (PMC12741057; doi:10.3389/fsurg.2025.1692887)
Supplement: Supplementary file 1 [file Supplementaryfile1.docx]

**Appendix - Supplementary Materials**

**A1. Bibliographic Search Syntax**

**Table S1.** Combination of free-text vocabulary and/or controlled terms (e.g., MeSH) used to identify studies in PubMed, Scopus, and Web of Science.

| **PubMed** | ("spondylosis"[Title/Abstract] OR "degenerative spinal disease*"[Title/Abstract] OR "back pain"[Title/Abstract] OR "spinal diseases*"[Title/Abstract] OR "degenerative intervertebral disks"[Title/Abstract] OR "spondylosis"[MeSH Terms] OR "spinal diseases/diagnostic imaging"[MeSH Terms]) AND ("arthrodes*"[Title/Abstract] OR "spinal stabili*"[Title/Abstract] OR "spinal fusion"[Title/Abstract] OR "interbody fusion"[Title/Abstract] OR "facet joint fusion"[Title/Abstract] OR "spinal fusion*"[Title/Abstract] OR "arthrodesis"[MeSH Terms] OR "arthrodesis"[MeSH Terms] OR "spinal fusion"[MeSH Terms] OR "lumbar vertebrae/surgery"[MeSH Terms] OR "spinal fusion/methods*"[MeSH Terms] OR "spinal diseases/surgery*"[MeSH Terms] OR "spinal fusion/instrumentation"[MeSH Terms]) AND ("Brantigan"[Title/Abstract] OR "brantigan score"[Title/Abstract] OR "bridwell interbody fusion grading system"[Title/Abstract] OR "fusion assessment*"[Title/Abstract] OR "fusion rate*"[Title/Abstract] OR "fusion classification*"[Title/Abstract] OR "fusion scale"[Title/Abstract] OR "fusion evaluation"[Title/Abstract] OR "radiologic assessment"[Title/Abstract] OR "tomography scanners, x ray computed"[MeSH Terms]) |
| --- | --- |
| **Scopus** | ( TITLE-ABS-KEY ( spondylosis OR "degenerative spinal disease" * OR back AND pain OR "spinal diseases" * OR "degenerative intervertebral disks" OR "spinal diseases/diagnostic imaging" ) AND TITLE-ABS-KEY ( arthrodes* OR "spinal stabili" * OR "spinal fusion" OR "interbody fusion" OR "facet joint fusion" OR "spinal fusion" * OR "arthrodesis" OR "spinal fusion" OR "lumbar vertebrae/surgery" OR "spinal fusion/methods" * OR "spinal diseases/surgery" * OR "spinal fusion/instrumentation" ) AND TITLE-ABS-KEY ( brantigan OR "brantigan score" OR "bridwell interbody fusion grading system" OR "fusion assessment" * OR "fusion rate" * OR "fusion classification" * OR "fusion scale" OR "fusion evaluation" OR "radiologic assessment" OR "tomography scanners, x ray computed" ) ) |
| **Web of science** | spondylosis OR "degenerative spinal disease" OR back pain OR "spinal diseases" OR "degenerative intervertebral disks" OR "spinal diseases/diagnostic imaging" (All Fields) and arthrodes OR "spinal stabili" OR "spinal fusion" OR "interbody fusion" OR "facet joint fusion" OR "spinal fusion" OR "arthrodesis" OR "spinal fusion" OR "lumbar vertebrae/surgery" OR "spinal fusion/methods"* OR "spinal diseases/surgery" OR "spinal fusion/instrumentation" (All Fields) and Brantigan OR "brantigan score" OR "bridwell interbody fusion grading system" OR "fusion assessment" OR "fusion rate" OR "fusion classification" OR "fusion scale" OR "fusion evaluation" OR "radiologic assessment" OR "tomography scanners, x ray computed" (All Fields) |

**A2. Regional trends in surgical practice**

Figure 1 illustrates the distribution of spinal fusion procedures across different anatomical regions. The data indicates that the lumbar spine is the most frequently treated region, accounting for 78.7% of all surgical interventions. Cervical spine procedures make up 16.1% of cases, while thoracic spine surgeries are the least common, representing only 5.3% of interventions. This distribution underscores the predominance of lumbar spine pathology as the main indication for surgical treatment. The high surgical volume in the lumbar region likely reflects its greater biomechanical load, wider range of motion, and higher susceptibility to degenerative changes compared with cervical and thoracic regions. These findings are consistent with existing literature highlighting the lumbar spine’s central role in weight-bearing and mobility, factors that predispose it to instability, disc degeneration, and other conditions frequently requiring fusion procedures.

*
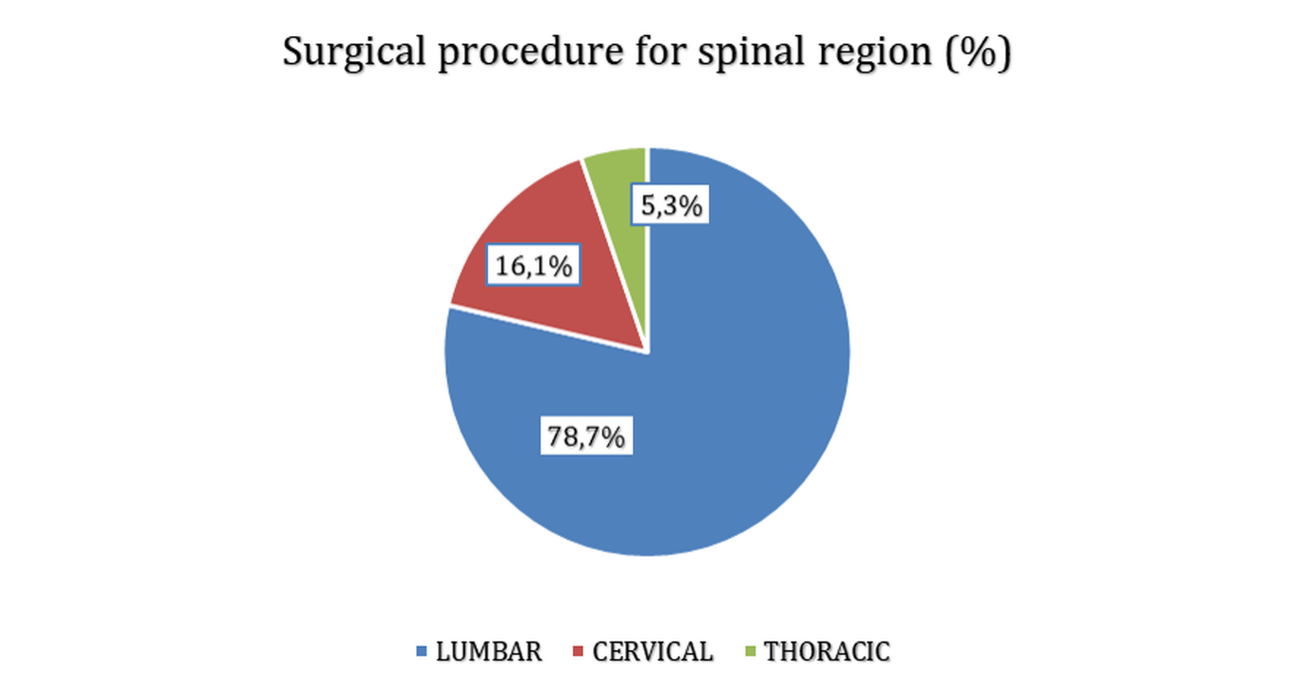
*

**Figure 1.** Distribution of spinal fusion surgeries by anatomical region.

Figures 2A-C provide a detailed analysis of clinical indications and corresponding surgical procedures across the cervical, thoracic, and lumbar spine regions, offering valuable insights into anatomical variability and surgical decision-making in spinal fusion.

Figure 2A illustrates the distribution of cervical spine pathologies leading to surgery. The most common indication is myelopathy (29.8%), followed by degenerative conditions (25.0%) and spondylolisthesis, spondylolysis, and spinal instability (18.3%). Less frequent indications include disc herniation and recurrence (5.8%), spinal stenosis (3.8%), and spinal deformities (2.9%). Regarding surgical interventions, ACDF is the predominant technique, accounting for 60.9% of cervical procedures. Other notable methods include ACCF (12.5%) and unspecified fusion techniques (11.7%). Less commonly employed procedures include OCF (0.8%), ACXF (2.3%), PCF (7.8%), and percutaneous approaches (3.9%). These data provide a comprehensive overview of clinical patterns and surgical preferences in cervical spine disorders.

Figure 2B summarizes thoracic spine pathologies and related surgical procedures. The most frequent indications include rare or specific conditions (34.3%), followed by degenerative disorders (20.0%), spondylolisthesis/spondylolysis/instability (20.0%), and spinal deformities (14.3%). Less common indications are spinal stenosis (2.9%) and disc herniation or recurrence (2.9%). In terms of surgical techniques, PLF is the most commonly performed procedure (21.4%), followed by TLIF (16.7%) and PLIF (14.3%). Other less frequently used approaches include OLIF (4.8%), LLIF (2.4%), and Minimally Invasive Surgery (cMIS) (7.1%). These findings reflect the complexity and heterogeneity of thoracic spine pathologies and their surgical management.

Figure 2C focuses on the lumbar spine, where degenerative disorders are the leading indication (40.5%), followed by spondylolisthesis/spondylolysis/instability (27.1%) and spinal stenosis (11.1%). Less frequent indications include spinal deformities (2.9%) and disc herniation or recurrence (3.6%). Surgical treatment in the lumbar region is dominated by TLIF (36.8%), followed by PLIF (19.1%) and unspecified fusion techniques (21.7%). Additional approaches include OLIF (8.5%), LLIF (7.2%), and ALIF (6.7%).

Taken together, Figures 2A-C offer a comprehensive overview of clinical and surgical trends across spinal regions, enhancing the understanding of contemporary spinal fusion practices. These findings underscore the importance of refining region-specific surgical strategies to optimize patient outcomes.


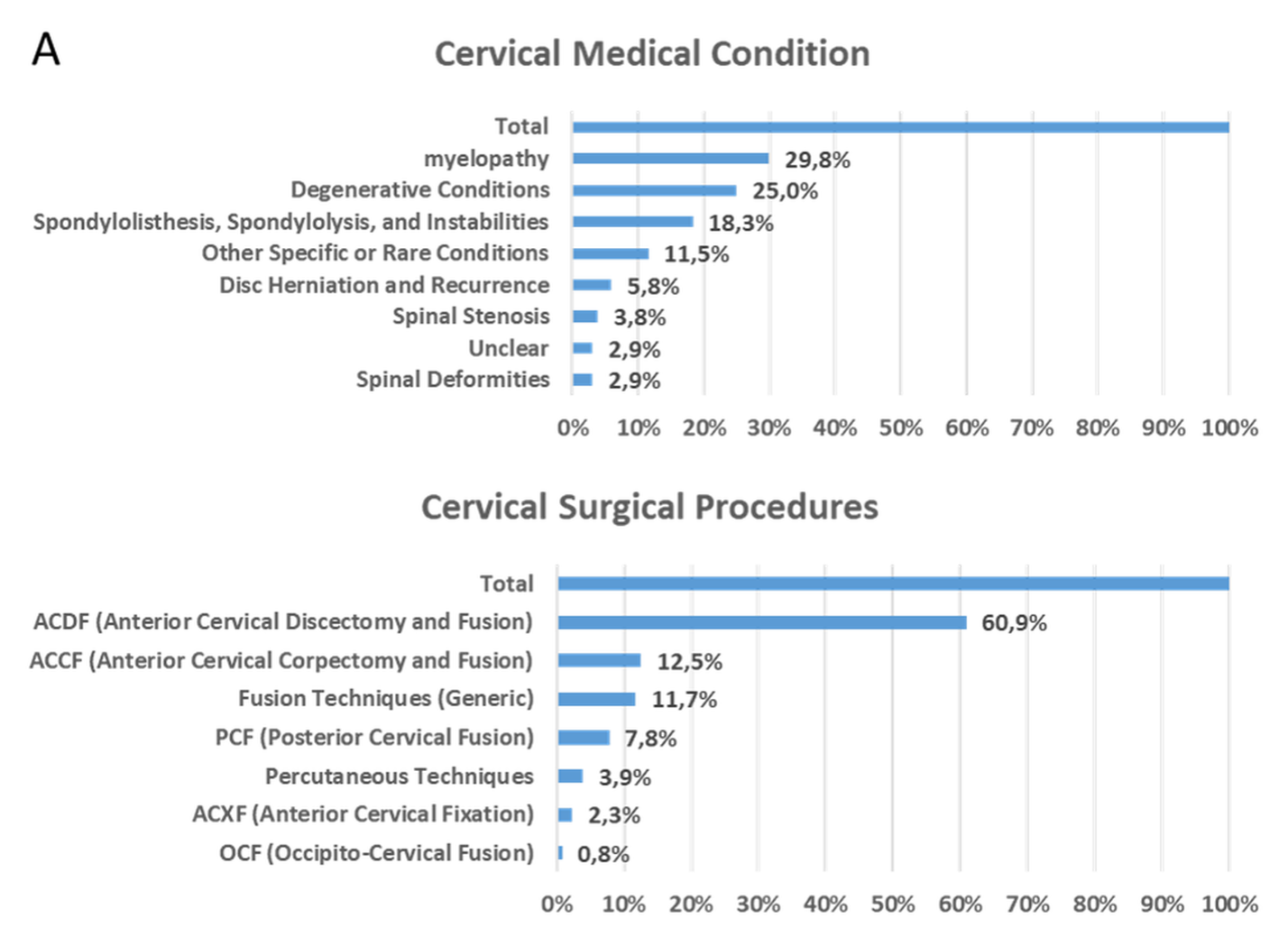


*
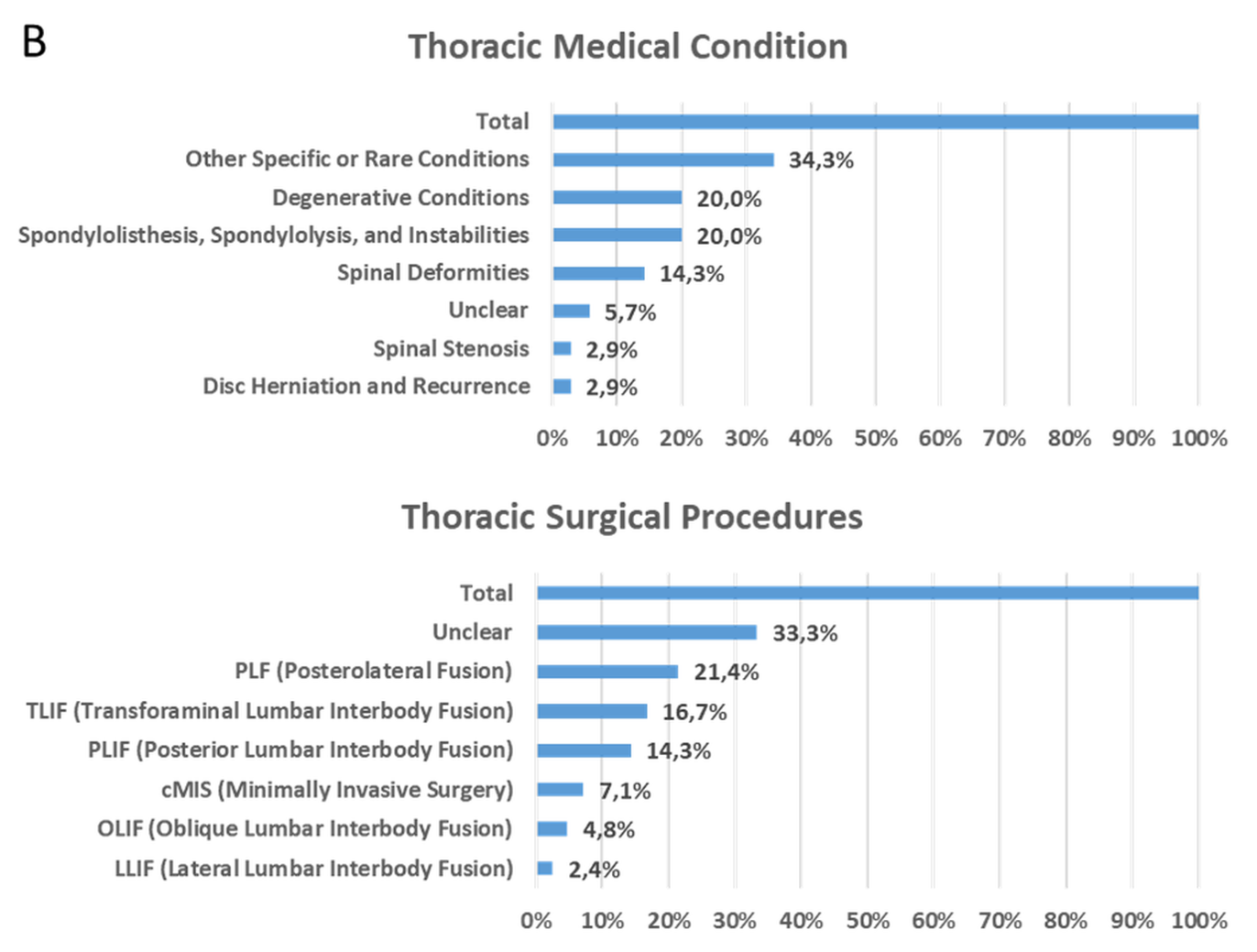
*

*
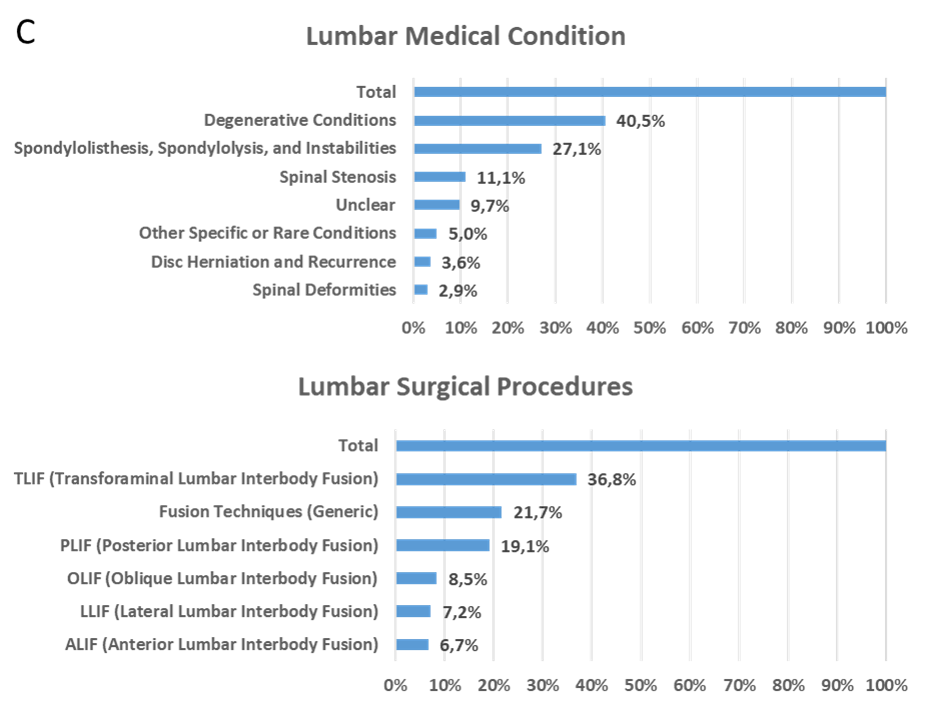
*

**Figure 2.** Surgical procedures and associated medical conditions in the A) cervical, B) thoracic, and C) lumbar spinal regions.
